# TWIK-Related Acid-Sensitive Potassium Channels (TASK-1) Emerge as Contributors to Tone Regulation in Renal Arteries at Alkaline pH

**DOI:** 10.3389/fphys.2022.895863

**Published:** 2022-05-20

**Authors:** Anastasia A. Shvetsova, Varvara S. Lazarenko, Dina K. Gaynullina, Olga S. Tarasova, Rudolf Schubert

**Affiliations:** ^1^ Faculty of Biology, M.V. Lomonosov Moscow State University, Moscow, Russia; ^2^ Physiology, Institute of Theoretical Medicine, Faculty of Medicine, University of Augsburg, Augsburg, Germany

**Keywords:** TASK-1 channel, vascular tone, renal artery, mesenteric artery, extracellular pH

## Abstract

**Aim:** TASK-1 channels are established regulators of pulmonary artery tone but their contribution to the regulation of vascular tone in systemic arteries is poorly understood. We tested the hypothesis that TASK-1 channel functional impact differs among systemic vascular beds, that this is associated with differences in their expression and may increase with alkalization of the extracellular environment. Therefore, we evaluated the expression level of TASK-1 channels and their vasomotor role in mesenteric and renal arteries.

**Methods:** Pulmonary, mesenteric and renal arteries from male Wistar rats were used for TASK-1 channel mRNA (qPCR) and protein content (Western blotting) measurements. The functional role of TASK-1 channels was studied by wire myography using the TASK-1 channel blocker AVE1231. In some experiments, the endothelium was removed with a rat whisker.

**Results:** Expression levels of both mRNA and protein of the TASK-1 channel pore-forming subunit were highest in pulmonary arteries, lowest in mesenteric arteries and had an intermediate value in renal arteries. Blockade of TASK-1 channels by 1 µM AVE1231 increased U46619-induced contractile responses of pulmonary arteries but did not affect basal tone and contractile responses to methoxamine of mesenteric and renal arteries at physiological extracellular pH (pHo = 7.41). At alkaline extracellular pH = 7.75 (increase of NaHCO_3_ to 52 mM) AVE1231 evoked the development of basal tone and increased contractile responses to low concentrations of methoxamine in renal but not mesenteric arteries. This effect was independent of the endothelium.

**Conclusion:** In the rat systemic circulation, TASK-1 channels are abundant in renal arteries and have an anticontractile function under conditions of extracellular alkalosis.

## Introduction

The TASK-1 (TWIK-related acid-sensitive potassium) channel is a member of the two-pore domain potassium channel (K2P) family. It is one of the dominant members among the K2P channel family in both pulmonary (Antigny et al., 2016) and systemic arteries (Gardener et al., 2004; Kiyoshi et al., 2006; Lloyd et al., 2009; Shvetsova et al., 2020). TASK-1 channels conduct background potassium currents in arterial smooth muscle cells, contributing to resting membrane potential (Goldstein et al., 2001; Gurney and Manoury, 2009). Therefore, TASK-1 channel activity counteracts Ca^2+^ influx through voltage-gated calcium channels and, consequently, suppresses vasocontraction (Tykocki et al., 2017). TASK-1 channels are sensitive to extracellular pH variations: acidification suppresses their activity, while alkalization, on the contrary, activates them (Duprat et al., 1997; Gurney et al., 2003).

An important vasomotor role of TASK-1 channels in the pulmonary circulation is beyond a doubt. Thus, TASK-1 channel closure induces depolarization of pulmonary arterial smooth muscle cells under hypoxic conditions, resulting in pulmonary vasoconstriction (Olschewski et al., 2006; Nagaraj et al., 2013). Therefore, TASK-1 channels can be opened at normoxia, suppressing vasocontraction. Notably, TASK-1 channel malfunction was shown to be associated with pulmonary arterial hypertension in rats and humans (Ma et al., 2013; Antigny et al., 2016; Lambert et al., 2019; Le Ribeuz et al., 2020).

However, there are only few studies addressing the functional impact of TASK-1 channels on the regulation of tone in systemic arteries. Alkalization of the extracellular solution or application of halothane [inhalational anesthetic activating TASK-1 (Patel et al., 1999)] led to hyperpolarization of arterial smooth muscle from rat aorta and mesenteric arteries (Gardener et al., 2004; Kiyoshi et al., 2006). Blockade of TASK-1 channels with the use of anandamide, methanandamide or bupivacaine eliminated the observed effect, indicating the involvement of TASK-1 channels in this hyperpolarization (Gardener et al., 2004; Kiyoshi et al., 2006). However, anandamide, methanandamide, bupivacaine, and halothane were shown to also affect other potassium channel families in the same concentration range (Van den Bossche and Vanheel, 2000; White et al., 2001; Hashiguchi-Ikeda et al., 2003; Martín et al., 2012) indicating their non-selectivity regarding TASK-1 channels. In addition, halothane can affect calcium currents (Buljubasic et al., 1992; Hüneke et al., 2001). Therefore, the limited number of studied vascular regions and the non-selectivity of the substances used necessitate a more detailed study of the vasomotor role of TASK-1 channels in systemic arteries.

For a long time, the absence of a relatively specific blocker of TASK-1 channels complicated the study of their functional role in the vasculature. However, the situation has changed when AVE1231, initially described as a Kv1.5 channel blocker, was shown to have much higher affinity for TASK-1 channels (Putzke et al., 2007; Kiper et al., 2015). With the use of AVE1231 (or A293 — an alternative name of the substance) a prominent anticontractile role of TASK-1 channels was demonstrated in pulmonary arteries of adult rats (Antigny et al., 2016). Accordingly, AVE1231-exposed rats were characterized by early hemodynamic signs of pulmonary hypertension (Antigny et al., 2016). On the contrary, intravenous AVE1231 administration did not affect systemic blood pressure of adult rats (Shvetsova et al., 2020). Mean arterial pressure of *Kcnk3*-mutated rats, characterized by TASK-1 channel dysfunction, was not changed as well (Lambert et al., 2019). However, it is unknown whether the impact of TASK-1 channels on the regulation of vascular tone differs between the pulmonary and the systemic circulation since direct comparative analyses of TASK-1 channel expression and its functional role in pulmonary and systemic arteries have never been performed, to the best of our knowledge. Importantly, the systemic effects of TASK-1 channel blockade/dysfunction may not occur if these channels are not functionally important in all, but only in some vascular regions. Moreover, a functional role of TASK-1 channels in systemic arteries may emerge only under specific conditions.

Therefore, in this study we tested the hypothesis that TASK-1 channel functional impact differs among systemic vascular beds, that this is associated with differences in their expression and may increase with alkalization of the extracellular environment. Mesenteric and renal arteries were chosen as the main objects of this study since the small intestine and the kidney receive up to 20% of cardiac output in the rat (Mcdevitt and Nies, 1976), contributing considerably to the regulation of systemic arterial pressure. To evaluate the functional impact of TASK-1 channels, we used AVE1231.

## Materials and Methods

### Animals

All experimental procedures in this study complied with the European Convention on the protection of animals used for scientific purposes (EU Directive 2010/63/EU) and were approved by Russian institutional committees on animal welfare (93-g, approval date 27.06.2019). Adult male Wistar rats (body weight 350–450 g) were obtained from the vivarium of the Institute of General Pathology and Pathophysiology (Moscow, Russia). The animals were provided with food and water ad libitum and housed in the animal unit of the Biological Faculty of the Lomonosov Moscow State University with a controlled temperature of 21°С–24°С and a 12-h light-dark cycle. Rats were killed under CO_2_ anaesthesia by decapitation.

### Wire Myograph Experiments

Pulmonary arteries of the left lung lobe (2nd-3rd branch orders of the pulmonary trunk), small mesenteric arteries (1st-2nd branch orders of the superior mesenteric artery) and interlobar renal arteries (2nd-3rd branch orders of the renal arteries) were chosen as the objects of this study. The arteries were carefully isolated, cleaned from surrounding tissues in physiological salt solution (PSS) for vessel dissection (PSS I, for composition of all solutions see section «Solutions»), cut into 2-mm-long segments, and mounted in a wire myograph (410A or 620M, DMT A/S, Denmark) to measure isometric force. In some experiments the endothelium was gently removed using a rat whisker right after mounting.

After mounting, PSS I was changed to PSS II and the myograph chambers were heated to 37°C while continuously bubbled with a 5% CO_2_ + 95% O_2_ gas mixture to maintain pH at 7.4. Data were recorded at 10 Hz using an analogue-to-digital converter (E14-140М, L-CARD, Russia) and the PowerGraph 3.3 software (DISoft, Russia). After heating the myograph, PSS II was changed to pre-heated (37°C) Ca-free PSS and the normalization procedure was performed. Considering the arterial pressure level in the pulmonary circulation, pulmonary arteries were stretched to d20 (the diameter that the vessel segments would have if rounded and held at a transmural pressure of 20 mmHg) (Chadha et al., 2012a; Antigny et al., 2016). Mesenteric and renal arterial segments were stretched to 0.9d100 (Mulvany and Halpern, 1977; Tarasova et al., 2003). Thereafter, Ca-free PSS was replaced by PSS II, which was further used throughout the whole or those part of the experiments performed at pH 7.4.

At the beginning of each experiment, arteries were exposed to an external solution containing 60 mM KCl to assess vessel viability. Then mesenteric and renal arteries were activated by the α_1_-adrenoceptor agonist methoxamine (10 μM). Since pulmonary arteries demonstrate weak contractile responses to methoxamine (Turnheim, 1971), they were activated by the thromboxane A_2_ receptor agonist U46619 (1 μM). To evaluate the functional state of the endothelium, acetylcholine (10 μM) was added on the top of the methoxamine—or U46619-induced contraction (1 or 0.1 μM, respectively). The presence or the absence of a relaxation response evidenced the integrity or removal of the endothelium, respectively. Each activation step was followed by at least 15 min of washout.

The experimental protocol included two sequential concentration-response relationships to U46619 (concentration range from 1 nM to 3 μM) or methoxamine (concentration range from 10 nM to 100 μM). The first relationship was started 20 min after the end of the activation procedure. This basic protocol was used in two series of experiments.

The first series of experiments aimed to compare the functional impact of TASK-1 channels in pulmonary, mesenteric and renal arteries under normal physiological conditions (PSS II was used throughout the experiment). After 15-min washout from the first concentration-response relationship, one segment of each artery was incubated for 20 min with the TASK-1 channel blocker AVE1231 (1 μM) and the other one with an equivalent volume of the solvent (DMSO, 5 μl per 5 ml myograph chamber). Then the second concentration-response relationship to the contractile agonist was obtained.

**FIGURE 1:**
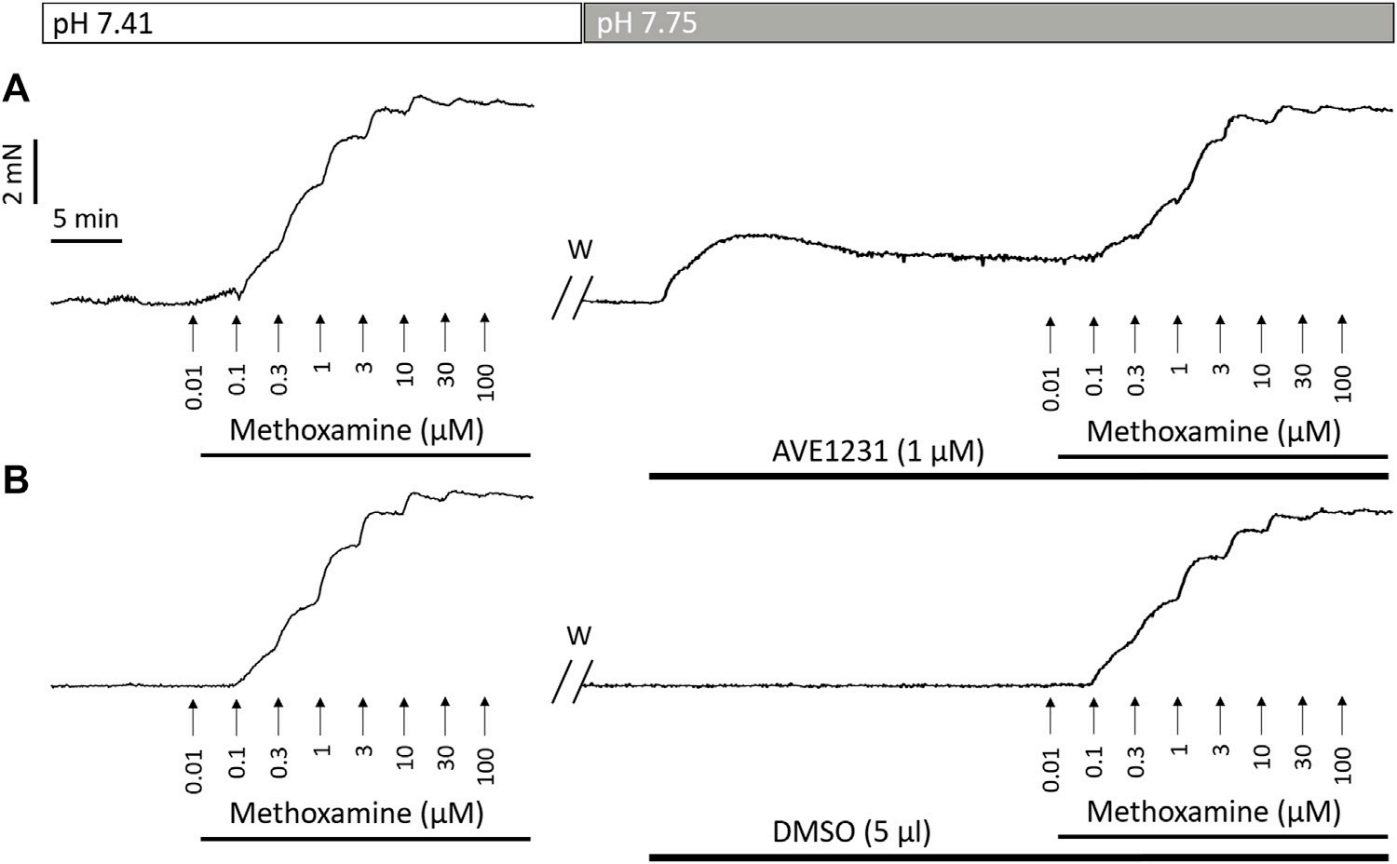
Recordings of contractile responses of two endothelium-denuded renal artery segments to methoxamine. The first concentration–response relationships [left panels in **(A,B)**] are similar for the two arterial segments. Application of the TASK-1 channel blocker AVE1231 (1 μM) increases basal tone as well as force values when methoxamine is added **(A)**, while incubation with an equivalent volume of the solvent (5 μl DMSO per 5 ml myograph chamber) has no such effects **(B)**. W, washout.

The second series of experiments aimed to evaluate the functional impact of TASK-1 channels on the regulation of vasocontraction in mesenteric and renal arteries under conditions of altered pH of the external solution (pHo). pHo was adjusted according to the Henderson-Hasselbach equation by manipulating the NaHCO_3_ concentration while maintaining a constant pCO_2_ by gassing the solution with 5% CO_2_ (Albuquerque and Leffler, 1998) (for detailed composition see section «Solutions»). Three experimental solutions were used: 1) acidic PSS (pHo 7.08, 13 mM NaHCO_3_), 2) normal PSS (pHo 7.41, 26 mM NaHCO_3_, the same as PSS II), and 3) alkaline PSS (pHo 7.75, 52 mM NaHCO_3_). After washout from the first concentration-response relationship, two segments of each arterial type were placed in acidic PSS, two segments—in normal PSS and two more segments in alkaline PSS. Five minutes later, AVE1231 (1 μM) was added to one segment in each pair and its solvent DMSO (5 μl per 5 ml chamber) to the other one for 20 min and the second concentration-response relationship was obtained (for details, see Figure 1). pHo was measured directly in the myograph chambers by a portable pH-meter (InLab Micro, Mettler Toledo, Germany/Switzerland). The recorded values were 7.08 ± 0.01, 7.41 ± 0.01 and 7.75 ± 0.01 for acidic PSS, normal PSS and alkaline PSS, respectively.

To calculate active force values, the force value at the fully relaxed state (obtained in Ca-free PSS) was subtracted from all recorded values. All active force values in the second concentration-response relationships (presented in Figures 1, 4, 5) were expressed as the percentage of the maximum active force developed in the respective first concentration-response relationship. Importantly, the first concentration–response relationships to the contractile agonist did not differ for arterial segments further treated with AVE1231 or solvent (Supplementary Figures S1–S3).

### Measurement of mRNA Expression Levels in Arterial Tissue by qPCR

Endothelium-intact pulmonary, mesenteric and renal arteries were isolated as described above and kept in RNA-later solution (Qiagen) at −20°C pending further procedures. Each sample included three pulmonary or mesenteric arteries or four renal interlobar arteries. Arteries were homogenized in RLT lysis buffer (Qiagen) containing 1% β-mercaptoethanol and proteinase K (0.2 mg/ml, MP Biomedicals, United States). RNA was extracted using the ExtractRNA kit (Evrogen, Russia) according to the manufacturer’s instructions and then processed with DNase I (Fermentas, 1.000 U/ml). The RNA concentration was measured by a NanoDrop 1000 (Thermo Scientific, United States), and then all samples were diluted to a concentration of 30 ng/μl. Reverse transcription was performed using the MMLV RT kit (Evrogen, Russia) according to the manufacturer’s manual. qPCR was run in the RotorGene6000 using qPCRmix-HS SYBR (Evrogen, Russia).

Primers used in this study were synthesized by Evrogen and had the following sequences: *Kcnk3* (encoding the TASK-1 pore-forming subunit gene, forward: TGT​CCA​TGG​CCA​ACA​TGG​T; reverse: GGA​AGA​AAG​TCC​AGC​GCT​CAT) and *Gapdh* (forward: CCA​TCA​AGG​ACC​CCT​TCA​TT; reverse: CAC​CAG​CAT​CAC​CCC​ATT​T). The *Kcnk3* mRNA expression level was calculated as E^−Ct^, where E is the primer efficiency and Ct is the cycle number at which the product fluorescence rose above the threshold level. E values were determined by dilution series and were close to 2.0. This *Kcnk3* expression level was normalized to the expression level for the housekeeping gene *Gapdh*, detected in the same sample. All values were expressed as the percentage of the mean value in pulmonary arteries.

### Measurement of Protein Abundance in Arterial Tissue by Western Blotting

Endothelium-intact arteries were isolated as described above, quickly frozen in liquid nitrogen and kept at −80°C till further analysis. Sample composition was the same to that for qPCR. Samples were homogenized in ice-cold SDS buffer supplemented with protease inhibitors cocktail (Roche) and phosphatase inhibitors (NaF 42 mg/ml, Na_3_VO_4_ 18 mg/ml), centrifuged at 16,900 g for 5 min; the supernatant was kept at −20°С till further analysis. Proteins were separated by SDS-PAGE and transferred to nitrocellulose membranes (Santa Cruz, United States) using the Trans-Blot Turbo transfer system (Bio-Rad, United States). Due to a large number of samples (*n* = 14 for each type of the artery), they were loaded on two gels (7 samples of mesenteric and renal arteries for each). The transfer was visualized with Ponceau S staining. Thereafter, the piece of membrane was cut out. The membrane was blocked with 5% nonfat milk (AppliChem, Germany) in TBS with 0.1% Tween 20 (TBSt). Then it was incubated overnight with rabbit polyclonal IgG antibodies against TASK-1 channels (Antigny et al., 2016; Lambert et al., 2019) (ab49433, Abcam, rabbit, 1:800 in TBSt with 5% BSA). The next day, the membrane was incubated with goat anti-rabbit IgG secondary antibodies (7074S, Cell Signalling, 1:10,000 in 5% milk in TBSt) for 1 h and visualized with SuperSignal West Dura Substrate (Thermo Fisher Scientific, United States) using ChemiDoc (Bio-Rad, United States). Thereafter, the membrane was stripped using stripping buffer (for composition see section «Solutions») since the loading control (GAPDH) had similar molecular weight as the protein of interest (TASK-1, approx. 40 kDa). The membrane was blocked again as described above and incubated overnight with antibodies against GAPDH (Abcam, 1:2000 in TBSt with 5% non-fat milk). The next day, it was incubated with horse anti-mouse IgG secondary antibodies (7076S, Cell Signaling, 1:5000 in 5% non-fat milk) for 1 h and visualized. Western blotting experiments were analyzed using the Image Lab software 6.0 (Bio-Rad, United States). In each membrane, individual values of TASK-1 channel protein content were normalized to respective GAPDH values and then the median ratio in mesenteric arteries was taken as 100%.

### Solutions

1) PSS for vessel isolation (PSS I), in mM: 145 NaCl, 4.5 KCl, 1.2 NaH_2_PO_4_, 1 MgSO_4_, 0.1 CaCl_2_, 0.025 EDTA, 5 HEPES.
2) PSS for myograph experiments (PSS II or normal PSS, pHo 7.41), in mM: 120 NaCl, 26 NaHCO_3_, 4.5 KCl, 1.2 NaH_2_PO_4_, 1.0 MgSO_4_, 1.6 CaCl_2_, 5.5 D-glucose, 0.025 EDTA, 5 HEPES; equilibrated with 5% CO_2_ in 95% O_2_.
3) Ca-free PSS, in mM: 120 NaCl, 26 NaHCO_3_, 4.5 KCl, 1.2 NaH_2_PO_4_, 1.0 MgSO_4_, 5.5 D-glucose, 0.1 EGTA, 5 HEPES; equilibrated with 5% CO_2_ in 95% O_2_.
4) Acidic PSS for myograph experiments (pHo 7.08), in mM: 133 NaCl, 13 NaHCO_3_, 4.5 KCl, 1.2 NaH_2_PO_4_, 1.0 MgSO_4_, 1.6 CaCl_2_, 5.5 D-glucose, 0.025 EDTA, 5 HEPES; equilibrated with 5% CO_2_ in 95% O_2_.
5) Alkaline PSS for myograph experiments (pHo 7.75), in mM: 94 NaCl, 52 NaHCO_3_, 4.5 KCl, 1.2 NaH_2_PO_4_, 1.0 MgSO_4_, 1.6 CaCl_2_, 5.5 D-glucose, 0.025 EDTA, 5 HEPES; equilibrated with 5% CO_2_ in 95% O_2_.
6) SDS-buffer: 0.0625 M Tris-HCl (pH 6.8), 2.5% SDS, 10% water-free glycerin, 2.47% dithiothreitol, 0.002% bromophenol blue supplemented with protease and phosphatase inhibitors (aprotinin 50 mg/ml, leupeptin 100 mg/ml, pepstatin 30 mg/ml, NaF 2 mg/ml, and Na_3_VO_4_ 180 mg/ml).
7) TBS: 50 mM Tris-HCl, 150 mM NaCl, pH 7.6.
8) TBS-T: TBS with 0.1% Tween.
9) Stripping buffer: 0.2 M glycine, 0.1% SDS, 1% Tween, pH 2.2.

### Drugs

Acetylcholine and methoxamine (all dissolved in H_2_O) were obtained from Sigma (United States). U46619 (dissolved in DMSO) was obtained from Cayman Chemical (United States). AVE1231 (dissolved in DMSO) was a kind gift of Sanofi.

### Statistical Data Analysis

Statistical analysis was performed using GraphPad Prism 7.0 (La Jolla, United States). The normality of the data distribution was tested using the Shapiro-Wilk test. Unpaired Student’s t-test, Mann-Whitney U test, one-way ANOVA or Repeated Measures ANOVA were used, as appropriate. Data are presented as mean and SEM or as median and the interquartile range depending on the type of data distribution; n represents the number of animals. The level of statistically significant differences was set as *p* < 0.05.

## Results

### Effects of TASK-1 Channel Blockade by AVE1231 on Contractile Responses of Pulmonary, Mesenteric and Renal Arteries Under Physiological Conditions

As the first step of our study, we evaluated the effect of TASK-1 channel blockade on contractile responses of arteries from the pulmonary and the systemic circulation under normal physiological conditions (endothelium-intact arteries, pHo 7.41). After normalization, the arteries were comparable in their inner diameter: 430 ± 22 µm for pulmonary arteries, 380 ± 8 µm for mesenteric arteries and 407 ± 11 µm for renal arteries (arterial segments were dissected from 6 rats, *p* > 0.05, one-way ANOVA).

**FIGURE 2:**
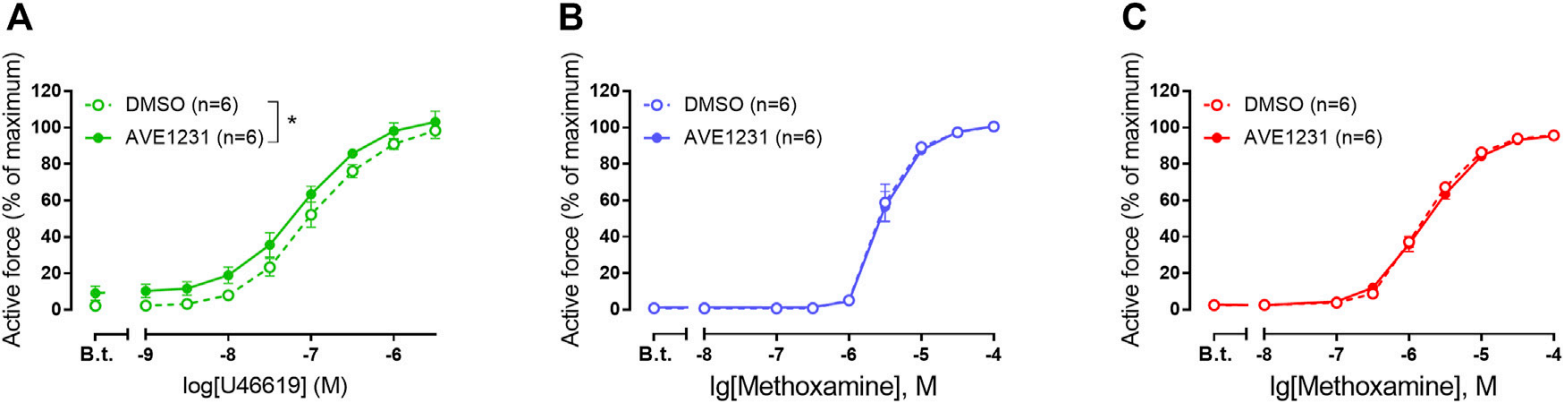
The effect of TASK-1 channel blockade on the contractile responses of rat pulmonary **(A)**, mesenteric **(B)** and renal **(C)** arteries. Concentration-response relationship to the thromboxane A_2_ receptor agonist U46619 **(A)** or the α_1_-adrenoceptor agonist methoxamine **(B,C)** in the presence of solvent (DMSO, 5 µl per 5 ml myograph chamber) or AVE1231 (1 µM). B.t.—basal tone (active force level before the first concentration of U46619 or methoxamine). Data are presented as mean ± SEM. *p < 0.05 (repeated measures ANOVA).

In our experiments, pulmonary arteries served as a positive control to correlate functioning of the TASK-1 channel blocker AVE1231 with a high TASK-1 channel expression level. As expected (Antigny et al., 2016), AVE1231 increased contractile responses of pulmonary arteries to U46619 (Figure 2A). However, it did not change the level of basal tone and contractile responses of mesenteric and renal arteries to methoxamine (Figures 2B,C). In addition, AVE1231 had no effect on U46619-induced contractions of renal arteries (Supplementary Figures S4). Therefore, a functional contribution of TASK-1 channels to the regulation of vasocontraction is not manifested in mesenteric and renal arteries under normal physiological external pH (Supplementary Table S1).

### TASK-1 Channel Abundance in Pulmonary, Mesenteric and Renal Arteries

In order to identify whether the differential functional impact of TASK-1 channels to the regulation of vasocontraction correlates with their abundance in the arterial wall, we compared mRNA and protein levels of the TASK-1 channel pore-forming subunit in pulmonary, mesenteric and renal arteries.

**FIGURE 3:**
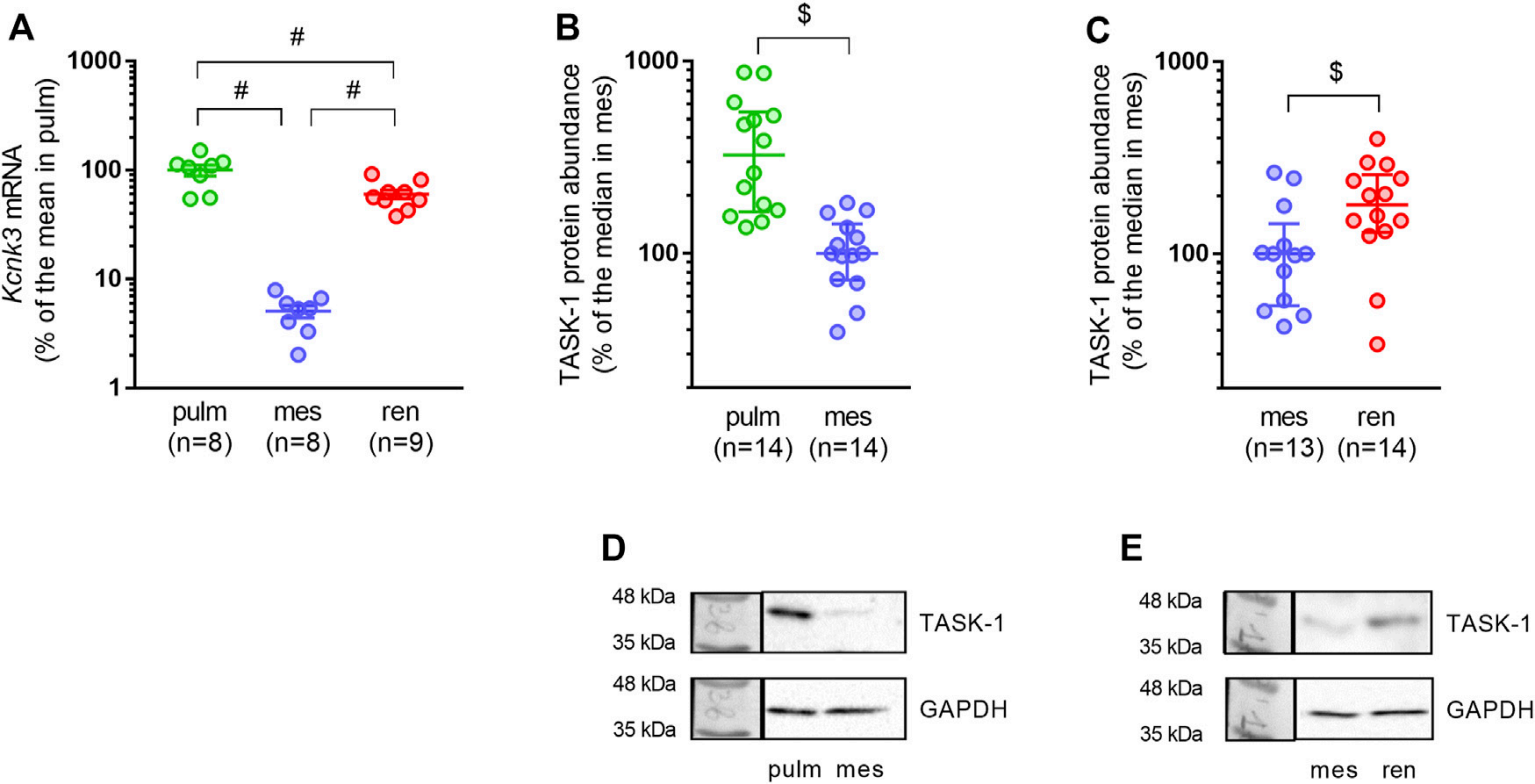
TASK-1 channel abundance differs in pulmonary, mesenteric and renal arteries. **(A)** Relative expression levels of *Kcnk3* pore-forming subunit mRNA in pulmonary, mesenteric and renal arteries. Data are normalized to *Gapdh* and then the mean value of *Kcnk3* mRNA in pulmonary arteries is taken as 100%. Data are presented as mean ± SEM. #*p* < 0.05 (One-Way ANOVA followed by Turkey’s post hoc analysis). **(B,C)** Protein content of the TASK-1 channel pore-forming subunit in pulmonary vs. mesenteric arteries **(B)** and in mesenteric vs. renal arteries **(C)**. **(D,E)** Representative Western blot images for pulmonary vs. mesenteric arteries **(D)** and mesenteric vs. renal arteries **(E)**. Data are normalized to GAPDH. The median value of TASK-1 channel protein content in mesenteric arteries is taken as 100%. Data are presented as median and interquartile range. $*p* < 0.05 (Mann-Whitney U test).

As expected, pulmonary arteries demonstrated the highest relative mRNA expression level of the TASK-1 channel pore-forming subunit (Figure 3A). In mesenteric arteries, TASK-1 channel mRNA content was almost 20 times lower compared to pulmonary arteries (Figure 3A). The TASK-1 channel mRNA expression level in renal arteries was surprisingly high: the mean value was 12 times higher than in mesenteric arteries and only 1.7 times lower than in pulmonary arteries (Figure 3A). Similarly, protein abundance of TASK-1 channels in mesenteric arteries was lower in comparison to both pulmonary and renal arteries (Figures 3B,C).

Therefore, high mRNA and protein abundance of TASK-1 channels in pulmonary arteries is associated with their prominent functional role as previously demonstrated (Olschewski et al., 2006; Antigny et al., 2016). The absence of TASK-1 channel impact at the functional level in mesenteric arteries correlates with their low expression level. Despite an absence of functional impact, TASK-1 channels are relatively highly expressed in renal arteries.

### Effects of Altered Extracellular pH on the Vasomotor Role of TASK-1 Channels in Mesenteric and Renal Arteries

In further experiments, we focused on the vasomotor role of TASK-1 channels in mesenteric and renal arteries at altered extracellular pH. It is known that extracellular acidification suppresses TASK-1 channel activity, while extracellular alkalization, on the contrary, activates them (Duprat et al., 1997; Gurney et al., 2003). Therefore, TASK-1 channels may reveal their vasomotor role under conditions of elevated extracellular pH, supposedly in renal arteries, characterized by a relatively high expression of the channel pore-forming subunit. To test this, the effects of AVE1231 were evaluated under acidic (7.08), normal (7.41) and alkaline (7.75) extracellular pH in mesenteric and renal arteries.

**FIGURE 4:**
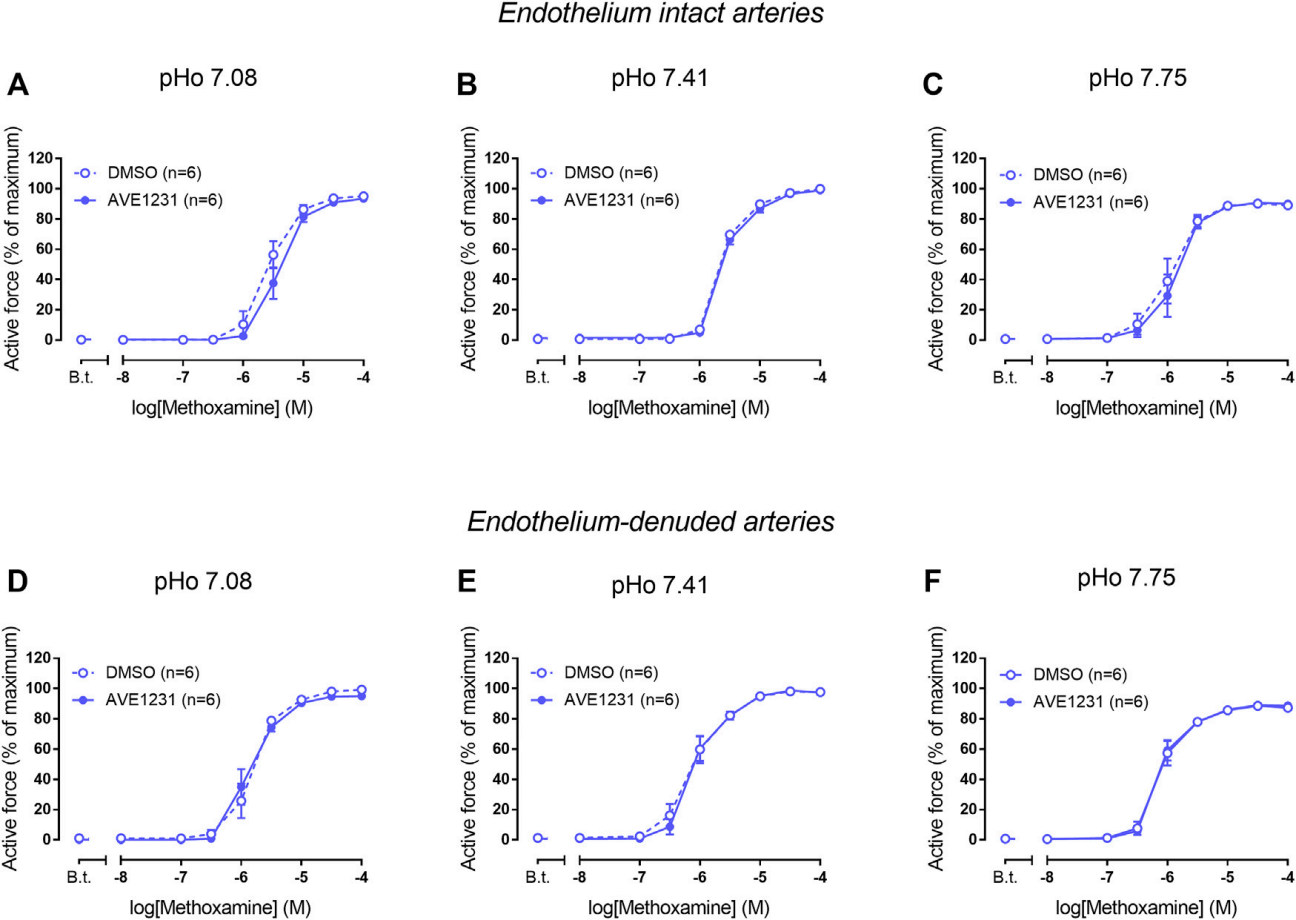
Effects of TASK-1 channel blockade on the contractile responses to methoxamine of endothelium-intact **(A–C)** and endothelium-denuded **(D–F)** mesenteric arteries at different extracellular pH (pHo). Concentration-response relationships to methoxamine in the presence of solvent (DMSO, 5 µl per 5 ml myograph chamber) or the TASK-1 channel blocker AVE1231 (1 µM) obtained in acidic [**(A,D)**, pHo 7.08], normal [**(B,E)** pHo 7.41] and alkaline [**(C,F)** pHo 7.75] PSS. B.t.—basal tone (active force level before the first concentration of methoxamine). Data are presented as mean ± SEM.

We did not observe any effect of AVE1231 on contractile responses of endothelium-intact mesenteric arteries at pHo 7.08 (Figure 4A). As observed in previous experiments, AVE1231 did not change basal tone and contractile responses of mesenteric arteries to methoxamine at normal pHo 7.41 (Figure 4B). In addition, AVE1231 did not alter contractile responses of mesenteric arteries at alkaline pHo 7.75 (Figure 4C). Similar results were obtained in experiments performed on endothelium-denuded mesenteric arteries (Figures 4D–F). Accordingly, AVE1231 has no effect on the sensitivity of arteries to methoxamine, as demonstrated by similar pD2 values (negative logarithm of EC50) of concentration-response relationships obtained for DMSO- or AVE1231-treated arterial segments, as well as on maximal active force (Supplementary Table S2).

**FIGURE 5:**
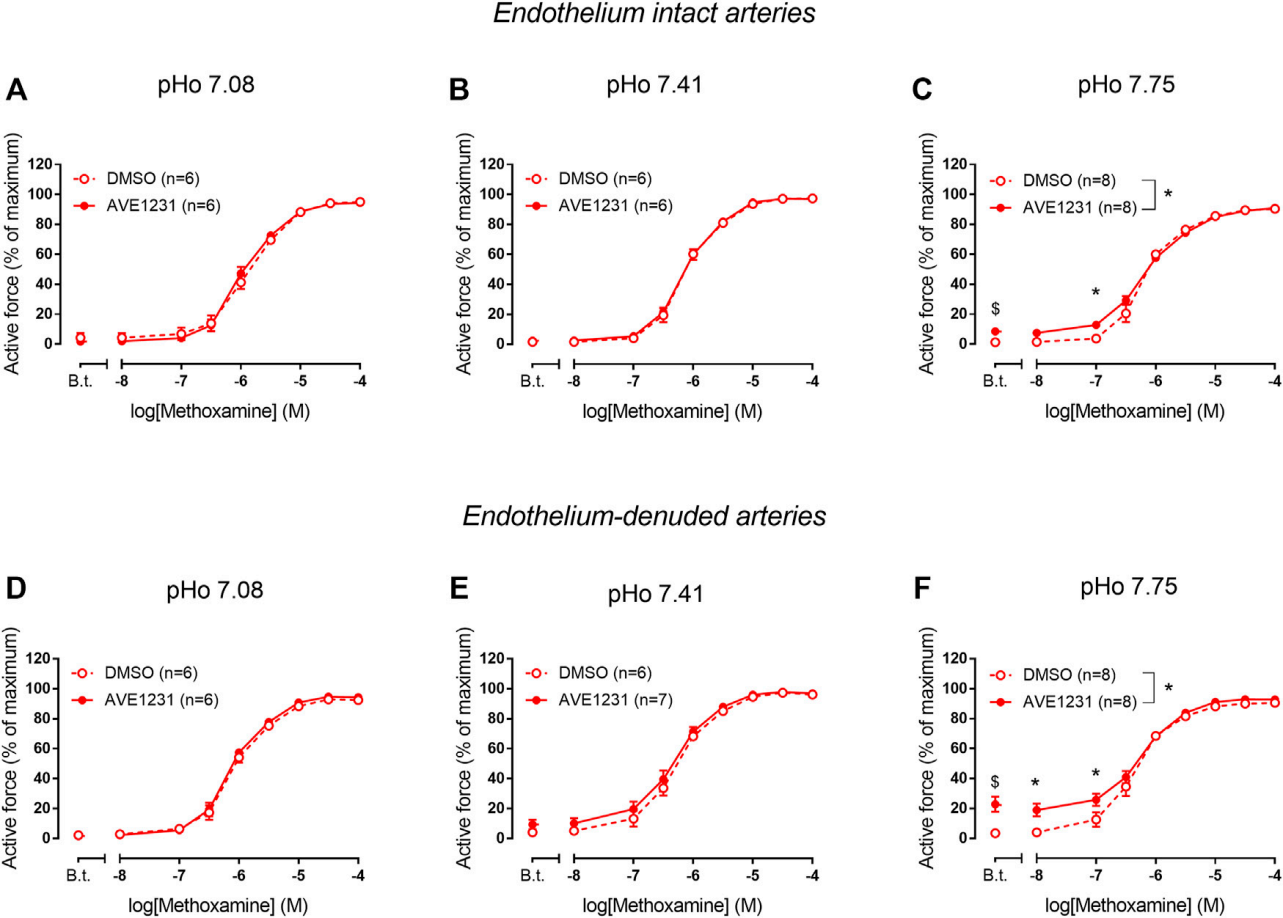
Effects of TASK-1 channel blockade on the contractile responses to methoxamine of endothelium-intact **(A–C)** and endothelium-denuded **(D–F)** renal arteries at different extracellular pH (pHo). Concentration-response relationships to methoxamine in the presence of solvent (DMSO, 5 µl per 5 ml chamber) or the TASK-1 channel blocker AVE1231 (1 µM) obtained in acidic [**(A,D)**, pHo 7.08], normal [**(B,E)**, pHo 7.41] and alkaline [**(C,F)**, pHo 7.75] PSS. B.t.—basal tone (active force before the first concentration of methoxamine). Data are presented as mean ± SEM. *p < 0.05 (repeated measures ANOVA with Sidak’s post hoc test). $*p* < 0.05 between basal tone values of AVE1231 and DMSO application (unpaired student’s t test).

In endothelium-intact renal arteries, TASK-1 channel blockade also did not affect basal tone and contractile responses to methoxamine at acidic and normal pHo (Figures 5A,B). However, at alkaline pHo AVE1231 caused the development of basal tone (Figure 5C) and increased active force at low concentrations of methoxamine while incubation with an equivalent volume of the solvent did not show such effects (Figures 1, 5C). At all pH levels studied, AVE1231 did not affect pD2 and E_max_ values of the concentration-response relationships (Supplementary Table S3). The effects of AVE1231 in renal arteries were independent of the endothelium since qualitatively similar effects were observed in endothelium-denuded arterial segments (Figures 5D–F, Supplementary Table S3). Therefore, TASK-1 channels exhibit an anticontractile role at the level of basal tone in renal arteries at alkaline pHo.

## Discussion

This study provides novel data on the expression and functioning of TASK-1 channels in systemic arteries of the rat. We showed that expression levels of both mRNA and protein of the TASK-1 channel pore-forming subunit were lower in systemic arteries in comparison to pulmonary arteries. At the same time, TASK-1 channel expression differs between the two studied systemic arteries: TASK-1 channel pore-forming subunit mRNA and protein contents were considerably higher in renal than in mesenteric arteries. For the first time, we studied the functional impact of TASK-1 channels in mesenteric and renal arteries with the use of AVE1231 that was initially described as a Kv1.5 channel blocker, but later demonstrated higher affinity for TASK-1 channels (Kiper et al., 2015). Importantly, in our previous study on rat arteries we obtained functional evidence using the Kv1.5 channel blocker DPO-1 that the effect of AVE1231 at the concentration used (1 µM) was selective for the TASK-1 channel (Shvetsova et al., 2020). In both systemic arteries, TASK-1 channels did not reveal an anticontractile role at physiologically normal pH. However, TASK-1 channels had an anticontractile function under conditions of extracellular alkalosis in renal but not mesenteric arteries, in accordance with the observed pattern of their expression. The effect of AVE1231 on alkalized renal arteries was manifested mainly at the level of basal tone.

### TASK-1 Channel Expression Differs Between Rat Mesenteric and Renal Arteries

TASK-1 channel expression has been demonstrated in pulmonary artery smooth muscle cells of rats (Gardener et al., 2004; Antigny et al., 2016), rabbit (Gurney et al., 2003) and human (Olschewski et al., 2006; Antigny et al., 2016). High TASK-1 channel abundance in pulmonary arteries as observed in our study, was expected since a prominent functional contribution of these channels to the regulation of pulmonary vascular tone was shown in a number of previous studies (Olschewski et al., 2006; Antigny et al., 2016). However, to the best of our knowledge, a comparative analysis of TASK-1 channel abundance between pulmonary and systemic arteries has never been performed.

Among arteries of the systemic circulation, presence of TASK-1 channel mRNA or protein was proven for the middle cerebral artery (Lloyd et al., 2009), saphenous artery (Shvetsova et al., 2020) and aorta of the rat (Kiyoshi et al., 2006). Further, the presence of TASK-1 channel expression was shown previously in mesenteric arteries (Gardener et al., 2004) and was reproduced in the present study. However, to the best of our knowledge, our study is the first to demonstrate TASK-1 channel expression in renal arteries, both at the mRNA and protein level. We demonstrated that TASK-1 channel mRNA and protein were highest in pulmonary arteries, lowest in mesenteric arteries, and had an intermediate value in renal arteries. Such differential TASK-1 channel expression suggests their different functional impact in the arteries studied.

### TASK-1 Channels do not Reveal a Vasomotor Role in Mesenteric and Renal Arteries at Physiological Normal pH

Although TASK-1 channel activity is potentiated by alkaline pH, the channels can conduct outward potassium currents even at a normal pH level of 7.3–7.4 (Gurney et al., 2003; Olschewski et al., 2006). Consequently, they are potentially capable to set a negative membrane potential preventing vasocontraction at physiological pH. Indeed, in our experiments, TASK-1 channel blockade with AVE1231 increased U46619-induced contractile responses of rat pulmonary arteries, reproducing the data by Antigny et al. (2016). Therefore, our data from pulmonary arteries demonstrate an anticontractile influence of TASK-1 channels in the pulmonary circulation at physiological normal pH, similar to data obtained in previous studies.

The contribution of TASK-1 channels to the regulation of vascular tone in systemic arteries is much less studied. Our data demonstrate that TASK-1 channel blockade by AVE1231 did not affect basal tone and contractile responses of mesenteric arteries at normal pH 7.41. Previously it was shown that blockade of TASK-1 channels by anandamide and bupivacaine led to a slight depolarization (approx. 3 mV) of smooth muscle from rat mesenteric arteries, indicating the involvement of TASK-1 channels in setting a negative membrane potential value (Gardener et al., 2004). However, these data should be interpreted with caution, since anandamide and bupivacaine can affect other potassium channels (Van den Bossche and Vanheel, 2000; White et al., 2001; Martín et al., 2012), and therefore are not selective TASK-1 blockers. Importantly, Gardener et al. did not measure the force of contraction simultaneously with the membrane potential and therefore it is not clear whether such a small depolarization can cause smooth muscle contraction. According to literature data and our previous experience, vessel contraction in response to such a small depolarization is unlikely since the membrane potential-contraction relationship in arterial smooth muscle is nonlinear (Mulvany et al., 1982; Neild and Kotecha, 1987; Shvetsova et al., 2019, 2020). In our experiments on saphenous arteries of adult rats the blockade of TASK-1 channels by AVE1231 (1 µM) or of Kv7 channels by XE991 (3 µM) depolarized arterial smooth muscle by about 7–12 mV, while it did not induce the development of basal tone (Shvetsova et al., 2019, 2020). Therefore, depolarization of arterial smooth muscle cells by approx. 3 mV induced by potential blockers of TASK-1 channels in the study by Gardener et al. (2004) seems to be rather subthreshold for smooth muscle contraction. Furthermore, the absence of an anticontractile influence of TASK-1 channels in mesenteric arteries correlates with their low mRNA and protein abundance.

The functional impact of TASK-1 channels on contractility of renal arteries has never been addressed before. Based on the relatively high expression level of TASK-1 channel pore-forming subunit mRNA and protein, we suggested a functional contribution of TASK-1 channels to the regulation of contraction in renal arteries. However, AVE1231 did not influence basal tone of renal arteries at pH 7.41. Neither methoxamine- nor U46619-induced contractile responses of renal arteries were changed by AVE1231, indicating that the absence of an anticontractile impact of TASK-1 channels in renal arteries at pH 7.41 is not related to the agonist used. It is known that the functional impact of a particular potassium channel may depend on the relative activities of other types of potassium channels expressed in the same cell. In other words, the functional role of one potassium channel may be masked by a potassium channel underlying a dominant potassium conductance. The latter was shown for the interplay between Kv7 and BKCa channels in saphenous arteries of young rats: Kv7 channels, being the dominant channel contributing to the negative feedback regulation of vasocontraction, functionally suppressed BKCa channels (Ma et al., 2020). Of note, a prominent contribution of Kv7 channels to the control of vascular tone was previously demonstrated in smooth muscle cells of rat renal arteries (Chadha et al., 2012b; Salomonsson et al., 2015). If Kv7 channels were the dominant potassium conductance in renal arteries, they may mask TASK-1 channel influence. This mechanism may also explain our observation that AVE1231 did not influence contractile responses of renal arteries under acidic conditions. However, for most of the potassium channels expressed in renal vessels, their functional activity at acidic pH is unknown. This hypothesis therefore needs to be clarified in future experiments. To conclude, our data demonstrate that TASK-1 channels are not functionally active in renal arteries under normal physiological or acidic conditions.

### TASK-1 Channels Counteract Vasocontraction of Renal but not Mesenteric Arteries at Alkaline pH

It is known that alkalization of the extracellular solution enhances the activity of TASK-1 channels (Duprat et al., 1997). Therefore, we suggested that TASK-1 channels in systemic arteries may reveal their vasomotor role under conditions of elevated extracellular pH.

Alkalization of the extracellular solution was shown to hyperpolarize arterial smooth muscle cells in rat mesenteric arteries by 3–4 mV (Gardener et al., 2004). Blockade of TASK-1 channels by anandamide or bupivacaine eliminated this effect of pH on membrane potential suggesting the involvement of TASK-1 channels in alkaline-induced hyperpolarization. In our study, TASK-1 channels did not reveal an anticontractile role in mesenteric arteries even under alkaline pH: AVE1231 did not affect basal tone and contractile responses to methoxamine. There could be several explanations for such a discrepancy between studies. Alkalization of the extracellular solution up to 7.75 as used in our study could activate TASK-1 channels to a lesser extent than alkalization to pH 8.4 performed by Gardener and colleagues. In addition, as mentioned above, anandamide or bupivacaine used in the study by Gardener et al. (2004) are non-specific blockers of TASK-1 channels (Van den Bossche and Vanheel, 2000; White et al., 2001; Martín et al., 2012) and there could be no role of TASK-1 channels in alkalization-induced vessel responses at all. Taken together, TASK-1 channels do not seem to regulate contractile responses of mesenteric arteries at alkaline pH 7.75.

In accordance with our qPCR and Western blot data, pointing to a quite high TASK-1 channel expression in renal arteries, we observed the development of basal tone and a higher level of active force at low concentrations of methoxamine in renal arteries under TASK-1 channel blockade at elevated pH of the extracellular solution. The effect of AVE1231 was manifested even after endothelium removal, which indicates that its effect is realized at the level of smooth muscle cells. Figure 1 clearly demonstrates that AVE1231 caused an increase in basal tone immediately after its application to the vessel, so that responses to the addition of methoxamine developed from an increased level of active force. Therefore, increased contractile responses at low concentration of methoxamine in AVE1231-treated segments (Figures 5C,D) were due to a higher basal tone, rather than the effect of metoxamine itself. This assumption is consistent with the observed similar sensitivities to methoxamine (pD2 values) in DMSO- and AVE1231-treated arterial segments.

In contrast to TASK-1 channels, blockade of some other potassium channels, such as Kv1, Kv7 and BKCa channels, induces a left-ward shift of concentration-response relationships and increases arterial sensitivity to methoxamine (Shvetsova et al., 2019). Notably, the α_1_-adrenoceptor agonist methoxamine induces membrane depolarization and Ca^2+^ influx, which can further activate Kv and BKCa channels with increasing agonist concentrations (Puzdrova et al., 2014; Shvetsova et al., 2019, 2020). Therefore, methoxamine can augment the functional availability of Kv and BKCa channels, which, in turn, provide negative feedback on methoxamine-induced contractions. Since TASK-1 channels are not sensitive to either membrane potential or intracellular Ca^2+^ (Goldstein et al., 2001), methoxamine does not increase their functional availability in smooth muscle cells. Notably, we observed a similar pattern of AVE1231 effects in saphenous arteries of young rats (Shvetsova et al., 2020). Thus, the functional impact of TASK-1 channels in systemic arteries is manifested mainly at the level of basal tone. The mechanism of the different pattern of TASK-1 channel blockade in pulmonary arteries is not currently clear and may be the subject of further research.

Thus, for the first time we demonstrated that TASK-1 channels counteract the development of basal tone in renal arteries at alkaline pH. Apparently, a prominent anticontractile influence of TASK-1 channels under conditions of alkalosis is especially important for the control of renal hemodynamics and renal function. It is known that kidneys may compensate metabolic alkalosis by removing the excess of bicarbonate anions from the organism (McKinney and Burg, 1977). TASK-1 channels may limit the renal blood flow decrease observed in alkalosis (Lockett, 1967). If small arteries proximal to the renal glomerulus were overcontracted under conditions of alkalosis, the intensity of glomerular filtration would be considerably reduced, decreasing the effectiveness of compensation. We suggest that the activation of TASK-1 channels in renal arteries during alkalosis prevents excessive contraction of the arteries, and thus helps to keep a relatively constant renal blood flow necessary for the implementation of the regulatory kidney function to maintain a constant pH.

## Limitations

Our study has two major limitations. The first of them concerns the Western blotting experiments. Notably, all commercially available antibodies against TASK-1 channels, including Abcam ab49433 used in this study, are not highly specific (Lambert et al., 2019). However, several studies demonstrated a correlation between TASK-1 channel protein expression assessed with the use of these antibodies and the functional impact of TASK-1 channels in pulmonary and systemic arteries (Antigny et al., 2016; Shvetsova et al., 2020). Similarly, functional and mRNA expression data obtained in the present study are in line with protein expression data. Therefore, protein expression data provide additional support for our conclusion of a greater role of TASK-1 channels in pulmonary and renal arteries in comparison to mesenteric arteries. The second limitation of the present study is that the evidence provided suggesting that TASK-1 channels affect vessel tone, especially at alkaline pH, was not supported by electrophysiological data. Concerning membrane potential measurements with sharp microelectrodes, it should be noted that renal arteries are not a suitable object for such measurements for reasons that are not yet clear. Thus, we got very low impalement success rates and a very high percentage of loss of impalements in renal arteries before the effect of AVE1231 reached steady state during membrane potential recordings. Patch-clamp experiments could also be considered. They could explore whether TASK-1 currents in isolated cells, e.g., smooth muscle cells, can in principle be activated by alkaline pH. Indeed, several studies have already reported the activation of TASK-1 currents by alkaline pH in smooth muscle cells (Gurney et al., 2003; Olschewski et al., 2006; Antigny et al., 2016). Importantly, smooth muscle cells are under completely different conditions after enzymatic isolation than cells in an intact vessel. Isolated smooth muscle cells are not subjected to cell-cell interactions, vessel wall stretch and other stimuli important for potassium channel functioning. Therefore, patch-clamp experiments do not answer the really crucial question of whether TASK-1 channels are active in smooth muscle cells at alkaline pH in intact renal arteries, i.e., under conditions that correspond to our functional myography experiments.

## Conclusion

In conclusion, our novel findings demonstrate that TASK-1 channel expression and functional impact differ between systemic and pulmonary arteries and, importantly, between two vascular regions of the systemic circulation — mesenteric and renal ones. We showed that TASK-1 channels do not reveal a vasomotor role in mesenteric and renal arteries under normal physiological pH, in contrast to pulmonary arteries, characterized by the highest abundance of TASK-1 channels. Our new data indicate a lack of TASK-1 channel impact on the regulation of vascular tone under normal physiological conditions in the systemic circulation. This suggestion is supported by data on the absence of an effect of TASK-1 channel blockade or its loss-of-function mutation on systemic arterial pressure of adult rats (Lambert et al., 2019; Shvetsova et al., 2020). At the same time, we observed that TASK-1 channels can emerge as contributors to an anticontractile effect in systemic arteries under specific conditions of elevated extracellular pH. We demonstrated that TASK-1 channels counteract vasocontraction of renal, but not mesenteric, arteries at alkaline pH. Importantly, the latter correlated with higher TASK-1 channel content in renal in comparison to mesenteric arteries. Probably, the prominent anticontractile role of TASK-1 channels in the renal arteries at alkaline extracellular pH may be an important mechanism contributing to the maintenance of a relatively constant renal blood flow and, therefore, compensatory function of kidneys under condition of alkalosis.

## Data Availability

The original contributions presented in the study are included in the article/Supplementary Material, further inquiries can be directed to the corresponding author.
