# Supplementary material for: TWIK-Related Acid-Sensitive Potassium Channels (TASK-1) Emerge as Contributors to Tone Regulation in Renal Arteries at Alkaline pH
Source: Front Physiol. 2022 May 20;13:895863. doi: 10.3389/fphys.2022.895863 (PMC9163564; doi:10.3389/fphys.2022.895863)
Supplement: Supplementary file 2 [file DataSheet1.PDF]

Original unprocessed images of Western blot membranes used in the article

Membrane 1

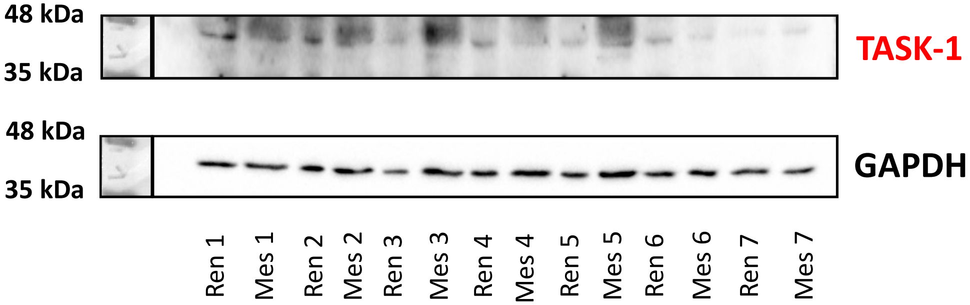

Membrane 2

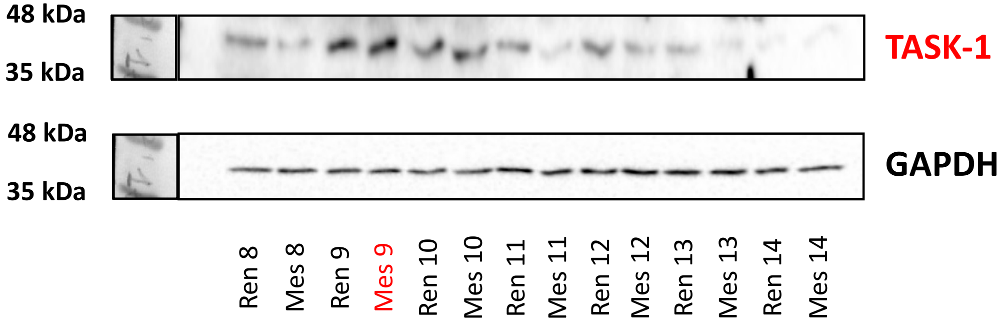

Ren – renal artery, mes – mesenteric artery; Sample «Mes 9» highlighted in red since was identified as outlier (by ROUT method)

Membrane 3

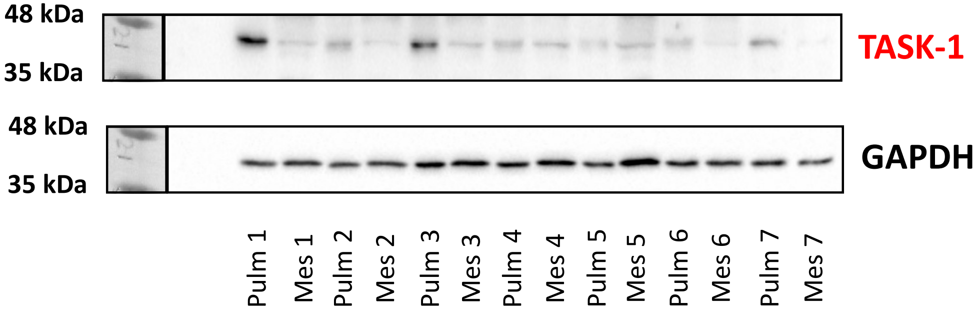

Membrane 4

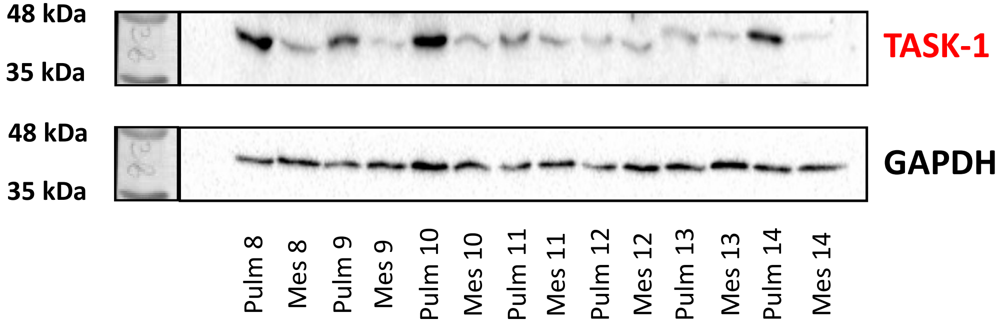

Pulm – pulmonary artery, mes – mesenteric artery
