# Supplementary material for: TWIK-Related Acid-Sensitive Potassium Channels (TASK-1) Emerge as Contributors to Tone Regulation in Renal Arteries at Alkaline pH
Source: Front Physiol. 2022 May 20;13:895863. doi: 10.3389/fphys.2022.895863 (PMC9163564; doi:10.3389/fphys.2022.895863)
Supplement: Supplementary file 3 [file DataSheet2.docx]

Supplementary Materials


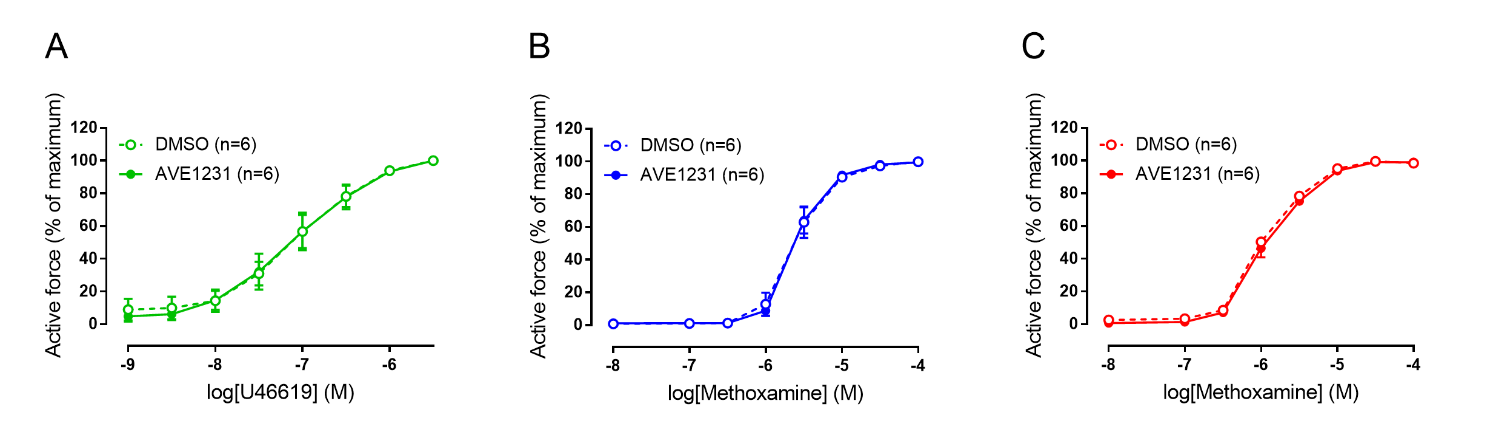


**Supplementary Figure 1.** First concentration-response relationships to the thromboxane A_2_ receptor agonist U46619 (A) or the α_1_-adrenoceptor agonist methoxamine (B, C) preceding the application of either blocker (AVE1231) or solvent (DMSO) to pulmonary (A), mesenteric (B) or renal arteries (C). Data are presented as mean ± SEM. Respective second concentration–response relationships are shown in Figure 2 in the main text of the manuscript.


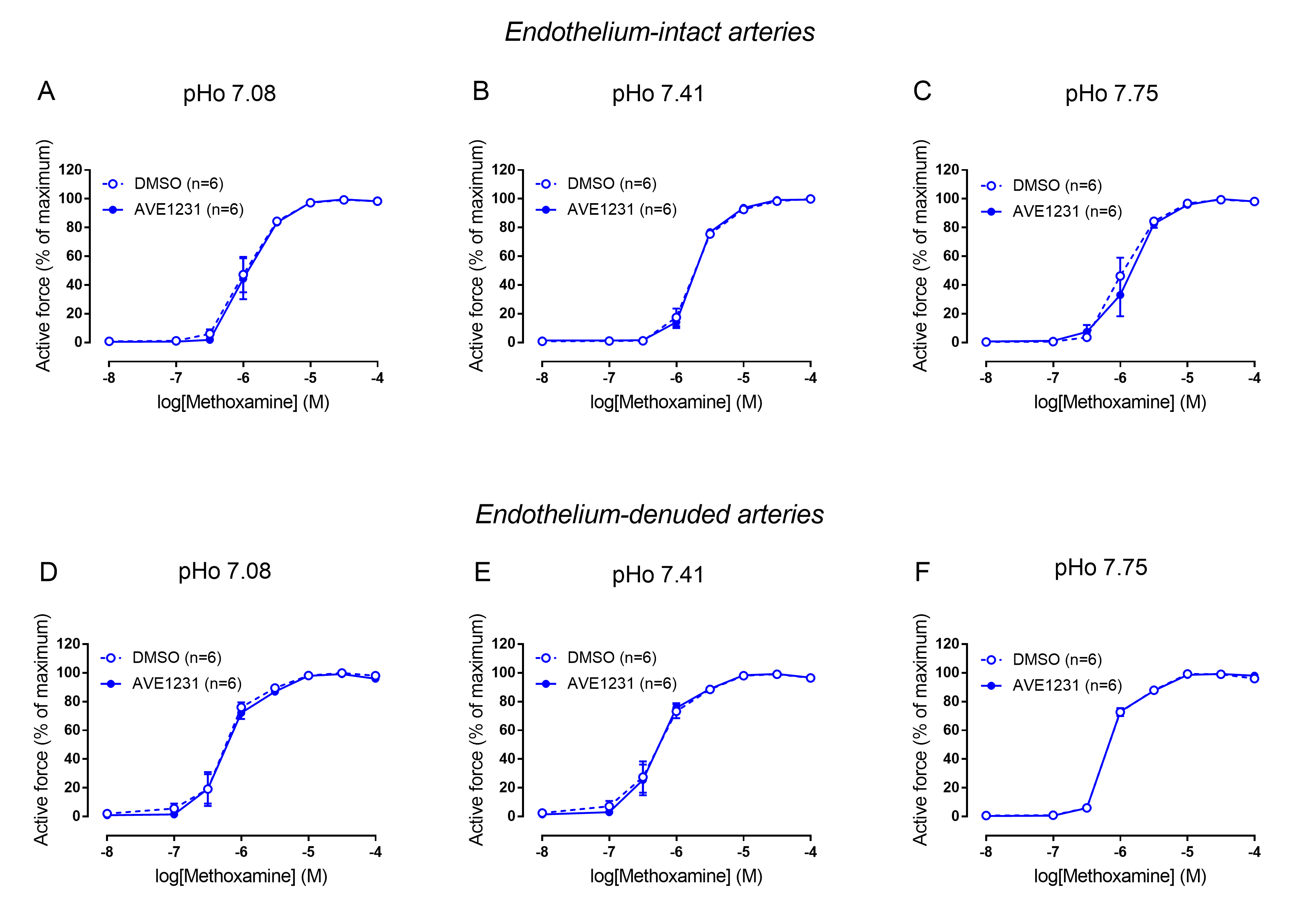


**Supplementary Figure 2.** First concentration-response relationships of mesenteric arteries to the α_1_-adrenoceptor agonist methoxamine preceding the extracellular pH (pHo) change and/or application of either blocker (AVE1231) or solvent (DMSO). Data are presented as mean ± SEM. Respective second concentration–response relationships are shown in Figure 4 in the main text of the manuscript.


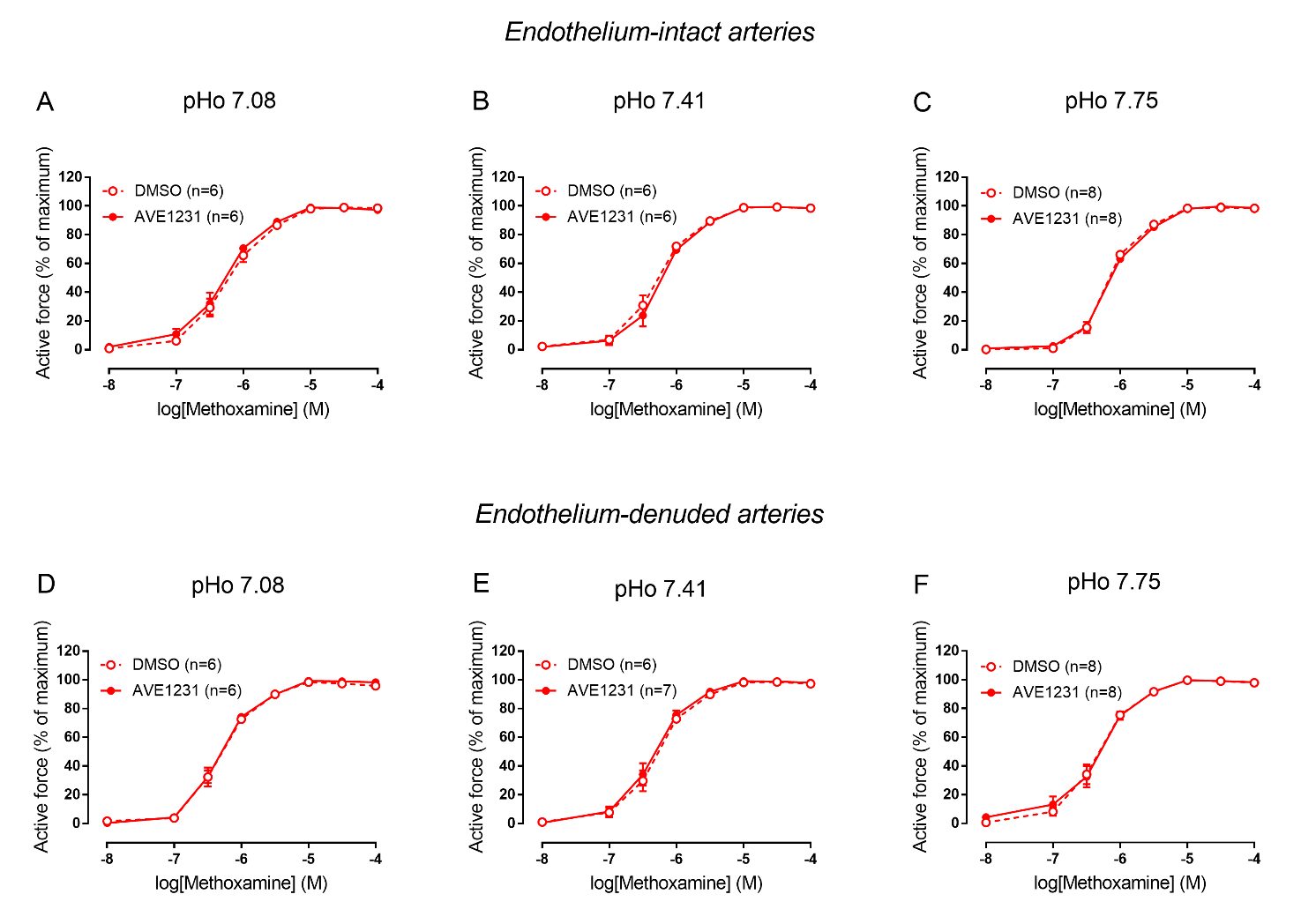


**Supplementary Figure 3.** First concentration-response relationships of renal arteries to the α_1_-adrenoceptor agonist methoxamine preceding the extracellular pH (pHo) change and/or application of either blocker (AVE1231) or solvent (DMSO). Data are presented as mean ± SEM. Respective second concentration–response relationships are shown in Figure 5 in the main text of the manuscript.


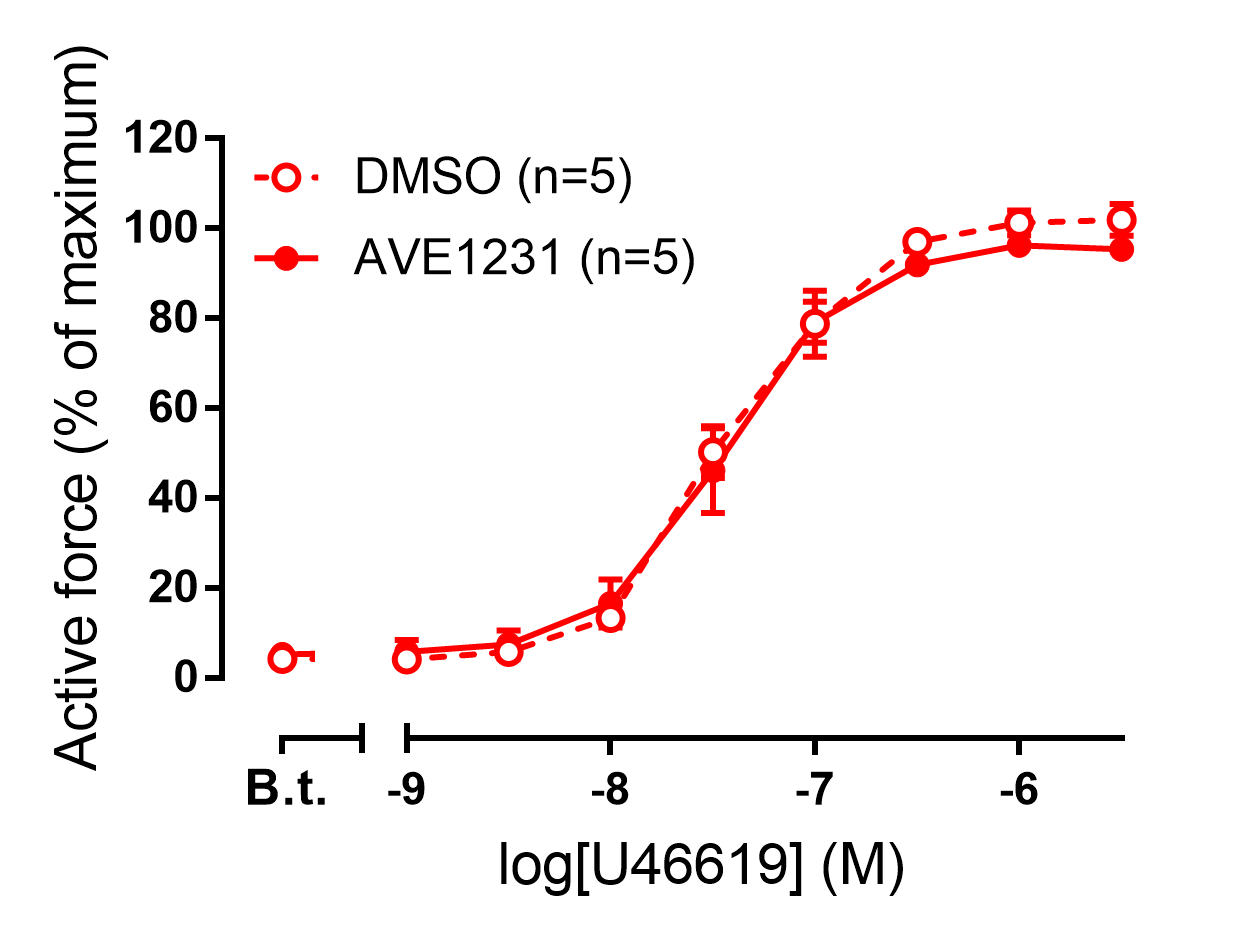


**Supplementary Figure 4.** Concentration-response relationships of rat renal arteries to the thromboxane A_2_ receptor agonist U46619 in the presence of solvent (DMSO, 5 µl per 5ml myograph chamber) or AVE1231 (1 µM). B.t. – basal tone (active force level before the first concentration of U46619). Data are presented as mean ± SEM.

**Supplementary Table 1**. pD2 and maximum active force (E_max_, mN) calculated for the concentration–response relationships to U46610 or methoxamine (**after the application** of either blocker or solvent) in pulmonary, mesenteric and renal arteries (respective concentration–response relationships are shown in Figure 2).

| Group of arteries | Pulmonary | | Mesenteric | | Renal | |
| --- | --- | --- | --- | --- | --- | --- |
|  | pD2 | E_max_ | pD2 | E_max_ | pD2 | E_max_ |
| DMSO (n=6;6;6) | 7.04±0.12 | 8.2±2.3 | 5.56±0.07 | 18.5±1.7 | 5.81±0.03 | 19.2±1.5 |
| AVE1231 (n=6;6;6) | 7.17±0.13 | 10.6±4.5 | 5.54±0.06 | 16.2±1.1 | 5.77±0.06 | 21.7±2.6 |

Data are presented as mean ± SEM

**Supplementary Table 2.** pD2 and maximum active force (E_max_) calculated for the concentration–response relationships to methoxamine (**after the application** of either blocker or solvent) in different series of experiments in endothelium intact and endothelium-denuded mesenteric arteries (respective concentration–response relationships are shown in Figure 4).

| pHo value | DMSO | | AVE1231 | |
| --- | --- | --- | --- | --- |
|  | pD2 | E_max_, mN | pD2 | E_max_, mN |
| *Endothelium-intact* | | | | |
| pHo 7.08 (n=6;6) | 5.61±0.10 | 20.2±0.9 | 5.42±0.08 | 18.4±1.2 |
| pHo 7.41 (n=6;6) | 5.64±0.03 | 19.6±1.7 | 5.61±0.02 | 17.9±1.9 |
| pHo 7.75 (n=6;6) | 5.97±0.14 | 17.4±1.3 | 5.88±0.12 | 18.9±1.4 |
| *Endothelium-denuded* | | | | |
| pHo 7.08 (n=6;6) | 5.80±0.09 | 19.5±2.3 | 5.87±0.09 | 18.1±1.5 |
| pHo 7.41 (n=6;6) | 6.10±0.12 | 17.6±1.2 | 6.09±0.08 | 17.2±1.2 |
| pHo 7.75 (n=6;6) | 6.11±0.08 | 17.2±1.5 | 6.10±0.06 | 16.8±1.3 |

Data are presented as mean ± SEM.

**Supplementary Table 3.** pD2 and maximum active force (E_max_) calculated for the concentration–response relationships to methoxamine (**after the application** of either blocker or solvent) in different series of experiments in endothelium intact and endothelium-denuded renal arteries (respective concentration–response relationships are shown in Figure 5).

| pH value | DMSO | | AVE1231 | |
| --- | --- | --- | --- | --- |
|  | pD2 | E_max_, mN | pD2 | E_max_, mN |
| *Endothelium-intact* | | | | |
| pHo 7.08 (n=6;6) | 5.86±0.04 | 19.5±1.6 | 5.97±0.05 | 20.9±1.1 |
| pHo 7.41 (n=6;6) | 6.13±0.04 | 19.2±1.9 | 6.12±0.05 | 17.4±1.3 |
| pHo 7.75 (n=8;8) | 6.20±0.06 | 16.1±1.3 | 6.14±0.05 | 18.6±2.0 |
| *Endothelium-denuded* | | | | |
| pHo 7.08 (n=6;6) | 6.07±0.05 | 16.0±2.6 | 6.11±0.03 | 12.4±1.6 |
| pHo 7.41 (n=6;7) | 6.28±0.05 | 15.4±1.7 | 6.28±0.06 | 13.4±1.2 |
| pHo 7.75 (n=8;8) | 6.37±0.06 | 12.7±1.1 | 6.23±0.03 | 13.2±1.7 |

Data are presented as mean ± SEM.
